# Bilateral Simultaneous Ureteroscopic (BS-URS) Approach in the Management of Bilateral Urolithiasis Is a Safe and Effective Strategy in the Contemporary Era—Evidence from a Systematic Review

**DOI:** 10.1007/s11934-017-0660-4

**Published:** 2017-02-17

**Authors:** Robert M. Geraghty, Bhavan P. Rai, Patrick Jones, Bhaskar K. Somani

**Affiliations:** 1 Department of Urology, University Hospital Southampton NHS Trust, Southampton, SO16 6YD UK; 2 James Cook University Hospital, Middlesbrough, UK; 3 Blackpool Teaching Hospital NHS Trust, Blackpool, UK

**Keywords:** Bilateral, Calculi, Laser, Review, Simultaneous

## Abstract

**Purpose of Review:**

Ureteroscopic treatment of urolithiasis has become safer and more effective in the modern era. With a rise in the incidence of bilateral urolithiasis, management dilemma of staged single-side ureteroscopy versus bilateral simultaneous ureteroscopy (BS-URS) is often debatable. This review evaluates the current evidence base for bilateral simultaneous ureteroscopic approach in the modern era.

**Recent Findings:**

A systematic review was conducted from 1990 to June 2016 including all English language articles reporting on outcomes of BS-URS for urolithiasis. Data was split into two periods: period 1: 2003–2012 and period 2: 2013–2016, and analysed using SPSS version 21. A total of 11 studies (491 patients) were identified from a literature search of 148 studies with mean age of 45 years and a male: female ratio of 2:1 and a mean operative time of 69 min (SD = ±15). The initial and final stone-free rate (SFR) was 87 and 93%, respectively. Post-operative stents were placed in 89% of patients with a mean hospital stay of 1.6 days (SD = ±0.5). Overall, there was a significant negative association between case volume (procedures per month) and complication rate (*p* = 0.045). Mean hospital stay was significantly longer in period 1 (1.9 days, SD = ±0.5) than period 2 (1.3 days, SD = ±0.3) and complications were also significantly higher in period 1 (47%) compared to period 2 (12%) (*p* < 0.001). There were six studies examining holmium laser (HL) lithotripsy and three examining pneumatic lithotripsy (PL). There were significantly more complications after PL than HL; however, their SFR was similar.

**Summary:**

Our review shows that the complication rates and hospital stay are significantly reduced in the contemporary data suggesting an improving trend in outcomes following BS-URS. Simultaneous bilateral ureteroscopic treatment of urolithiasis is safe and effective in the modern era. Safety is increased in centers with increased number of procedures performed and with laser lithotripsy.

## Introduction

The evolution of sophisticated miniature ureteroscopes and holmium laser technology has significantly reduced morbidity associated with ureteroscopy (URS). Evidence from contemporary literature suggests complications between 2.4 and 8.6% with URS [1, 2]. Ureteroscopy is increasing becoming the approach of choice in complex endourological management [2–4, 5•]. Despite this, the management of bilateral urolithiasis is a subject of much contention and debate amongst endourologists worldwide. Staged single-side URS is often employed in preference to a bilateral simultaneous ureteroscopic (BS-URS) approach. The concerns over BS-URS are due to higher morbidity in comparison with a staged approach. However the potential benefits of BS-URS include single anesthetic session and potentially reduced cost associated with treatment. In an era of austerity these are important considerations while making surgical decisions [6–8]. A recent systematic review of seven studies evaluating BS-URS did report an overall stone-free rate (SFR) of 90% suggesting it was a feasible strategy. However, they did report an overall complication rate of nearly 50%, although majority of them were Clavien score ≤ II [9]. In this updated review, we aim to evaluate the following:

1. Feasibility, effectiveness and safety of BS-URS approach
2. Outcomes of bilateral simultaneous ureteroscopic BS-URS approach in a contemporary era
3. Factors that predict complications of BS-URS approach

## Methods and Materials

### Evidence Acquisition: Criteria for Considering Studies for This Review

Inclusion criteria:

1. All articles written in the English language
2. Studies reporting on outcomes following BS-URS for urolithiasis
3. Patients of any age

Exclusion criteria:

1. Studies reporting on outcomes of BS-URS for non-urolithiasis indication such as malignancy
2. Studies with <10 patients

### Search Strategy and Study Selection

The systematic review was performed according to the Cochrane Review and the preferred reporting items for systematic reviews and meta-analyses (PRISMA) guidelines. The search strategy was conducted to find relevant studies from Ovid Medline without revisions (1996–July 2016), Cochrane Library (2016), CINAHL (1990–July 2016), Clinicaltrials.gov, Google Scholar and individual urologic journals.

Terms used included ‘bilateral’, ‘simultaneous’, ‘synchronous’, ‘ureteroscopy’, ‘ureterorenoscopy’, ‘calculi’, ‘stones’ and ‘urolithiasis’. Boolean operators (AND, OR) were used to refine the search.

The search was limited to English language articles between 1996 and July 2016. Authors of the included studies were contacted in the case of data not being available or clear. Level of evidence was assessed using the recommendations set out by the Centre for Evidence-Based Medicine [10].

**Fig. 1:**
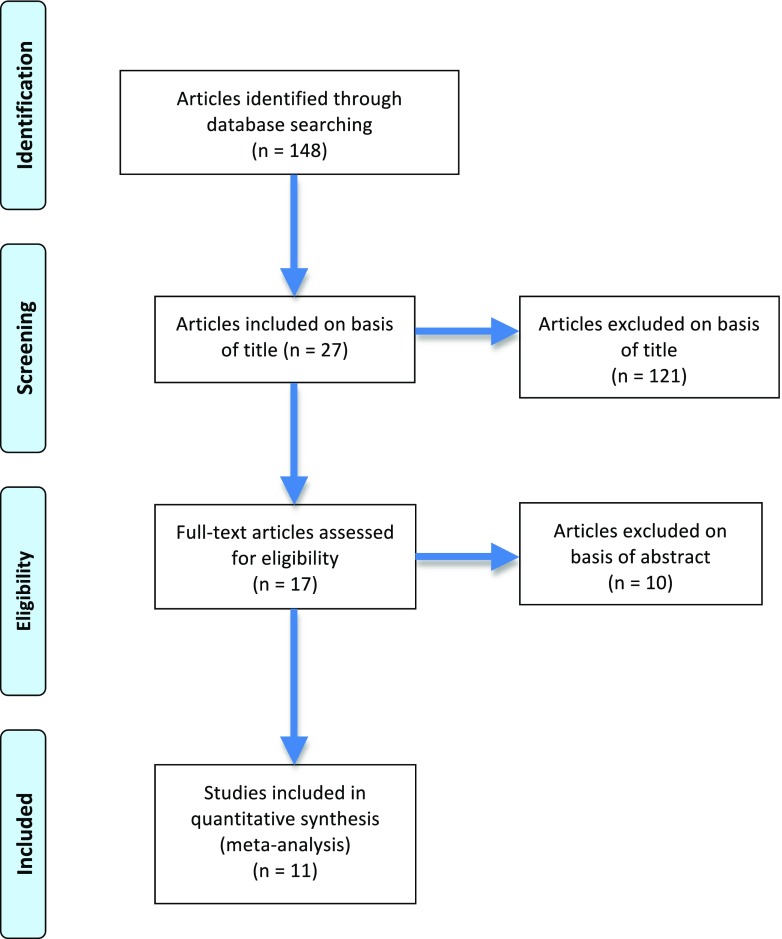
PRISMA flow diagram for article selection process

Two reviewers (RG and BS) independently identified all studies that appeared to fit the inclusion criteria [11–21] (see Fig. 1).

### Data Extraction and Analysis

The following variables were extracted from each study: year of publication, study period, number of patients/renal units included, operative time, initial and final SFR, lithotripsy fragmentation device, post-operative stent use, stone size (mm), cumulative stone diameter (CSD) (mm) and complications. Complications were graded using the Clavien Dindo classification [9] in all studies. Case volume was calculated as procedures per month during the study period as reported in the individual studies. For subanalysis, the data was divided into historical and contemporary studies by splitting the collated data into Period 1 (2003 to 2012 inclusive) and Period 2 (2013 to July 2016 inclusive) [22••].

Data was collated using Microsoft Excel (version 12.2.4) and analyzed using SPSS (version 21). Chi square test was used for dichotomous data and independent *T* test for continuous data.

### Outcome Measures

Primary outcomes:

1. Operative time;
2. Proportion of patient requiring insertion of ureteric stent
3. Stone-free rates (SFRs)
4. Hospital stay
5. Complication Rates

Secondary outcomes:

1. Compare outcomes between studies 2003–2012 (period 1) [22••] vs. 2013–2016 (period 2)
2. Evaluate predictors of complications.

## Results

### Literature Search and Included Studies

Our literature search produced 148 titles of which 131 articles were excluded due to non-relevance based on the title and abstract (see Fig. 1). Six further papers were excluded upon reading the full manuscript leaving 11 papers, which were included in the study [11–21]. Most studies were retrospective in nature and were no randomization or control groups in these studies (see Table 1). All studies reported on BS-URS and associated variables mentioned in the data extraction section.

**Table 1:** Study characteristics

Study	Type	LOE	URS
Hollenbeck, 2003 [11]	Unclear	3	Semi (6.9F)/Flexi (7.5F) + HL
Darabi, 2005 [12]	Unclear	3	Semirigid (unclear) + lithotripsy ND (8F Wolf or 10.5F Storz)
El-Hefnawy, 2011 [13]	Retrospective	3	Semirigid + PL + HL (8F/10F Wolf)
Mushtaque, 2012 [14]	Unclear	3	Semirigid + PL (7.8F)
Gunlusoy, 2012 [15]	Unclear	3	Semirigid (8F/10F) + PL
Huang, 2012 [16]	Retrospective	3	Flexi + HL (URF-P5 Olympus)
Isen, 2012 [17]	Unclear	3	Semirigid + PL (8F/9.8F Wolf)
Atis, 2013 [18]	Retrospective	3	Semi/Flexi + HL
Alkan, 2014 [19]	Retrospective	3	Flexi + HL (URF-P5 Olympus/Wolf)
Drake, 2015 [20]	Retrospective	3	Semirigid (6.5 F)/Flexi (Storz) + HL
Bansal, 2016 [21]	Retrospective	3	Flexi + HL

*LOE* level of evidence, *HL* holmium lithotripsy, *PL* pneumatic lithotripsy, *Flexi* flexible ureteroscope

The type of ureteroscope used varied in these studies. Five studies used semirigid ureteroscopes only [12–15, 17], two studies used flexible ureteroscopes only [16, 19] and the other four studies used a combination of semirigid and flexible ureteroscopes [11, 18, 20]. (see Table 1).

### Patient, Stone and Operative Characteristics

Overall there were 491 patients included in the review. Ten studies reported on the male to female ratio, which overall had a moderate male preponderance (2:1). The overall mean age was 44.7 years (SD = ±4.7 years). Stone size was reported in all but one study [12]. Mean stone size across these studies was 15.3 mm (SD = ±6.5 mm) (see Table 2).

**Table 2:** Patient and stone demographics

Study	Patients, *n*	M:F	Mean age, years (SD/range)	Mean cumulative stone diameter, mm (SD/range)
Hollenbeck, 2003 [11]	23	ND	52 (±14.9)	16.1 (±11.7)
Darabi, 2005 [12]	19	10:13	ND (4–78)	ND
El-Hefnawy, 2011 [13]	89	68:21	49 (13–74)	ND
Mushtaque, 2012 [14]	60	38:22	ND (11–60)	ND (5–20)
Gunlusoy, 2012 [15]	55	37:18	46.1 (22–81)	10.7 (±4.2, 5–21)
Huang, 2012 [16]	25	13:12	49.8 (28–69)	24 (±5, 17–37)
Isen, 2012 [17]	41	17:24	41.2 (28–76)	8.8 (7–16)
Atis, 2013 [18]	42	28:14	39.2 (±14.2)	24.09 (±6.37)
Alkan, 2014 [19]	42	28:14	40.1 (±10.8)	30.0 (±15.4, 10–85)
Drake, 2015 [20]	21	8:13	46 (22–76)	21 (4–63)
Bansal, 2016 [21]	74	50:24	39.2 (±15.2)	11.7 (±2.4)

*SD* standard deviation, *ND* not documented

Overall six studies [11, 16, 18–21] treated bilateral renal stones, three studies treated renal stones with contralateral ureteric stones [11, 19, 20] and eight studies [11–15, 17, 19, 20] treated bilateral ureteric stones (see Tables 3 and 4).

**Table 3:** Distribution of stones treated and stone-free rates (SFRs) of bilateral ureteric and renal stones

Study	Renal only, *n*	Renal/ureteric, *n*	Ureteric only, *n*	Ureteric SFR (%)	Renal SFR (%)
Hollenbeck, 2003 [11]	15	4	4	100	63
Darabi, 2005 [12]	–	–	38	84.2	ND
El-Hefnawy, 2011 [13]	–	–	178	95.5	ND
Mushtaque, 2012 [14]	–	–	120	85	ND
Gunlusoy, 2012 [15]	–	–	110	94.5	ND
Huang, 2012 [16]	128	–	–	ND	88.5
Isen, 2012 [17]	–	–	82	98.7	ND
Atis, 2013 [18]	84	–	–	ND	97.6
Alkan, 2014 [19]	47	37	4	ND	ND
Drake, 2015 [20]	12	11	2	100	75
Bansal, 2016 [21]	148	–	–	ND	ND
Total	434	52	538	94.0	81.0

*ND* Not documented, *n* number of patients

**Table 4:** Operative demographics

Study	Op time, min (SD/range)	Stent insertion, *n* (%)
Hollenbeck, 2003 [11]	90 ± 46	18 (75)
Darabi, 2005 [12]	ND	Unclear
El-Hefnawy, 2011 [13]	ND	78 (87.6)
Mushtaque, 2012 [14]	40–120	39 (65)
Gunlusoy, 2012 [15]	59 ± 21 (21–100)	96/110 units (87.3)
Huang, 2012 [16]	81.2 ± 25 (42–137)	25 (100)
Isen, 2012 [17]	58.4 (36–81)	41 (100)
Atis, 2013 [18]	51.08 (±15.22)	42 (71.4%—bilateral, 28.6%—unilateral)
Alkan, 2014 [19]	89.1 (±35.7)	36 (85.7)
Drake, 2015 [20]	70 (35–129)	25 (100) [7 unilateral, 18 bilateral]
Bansal, 2016 [21]	51.08 (±15.22)	65 (87.83)

*ND* not documented, *SD* standard deviation

### Definition of Stone-Free Status

There was variation in how ‘stone free’ status was defined amongst included studies. Four studies defined it as fragments <4 mm [17–19, 21]. Drake et al. [20], Huang et al. [16] and Gunlusoy et al. [15] defined stone free ≤2 mm, <1 mm and no stones, respectively. The rest did not specify how stone-free status was defined (see Table 5). Imaging modality and the time duration between intervention and imaging is also demonstrated in Table 5.

**Table 5:** Post-operative assessment

Study	SFR definition	Follow-up imaging to evaluate stone-free status	Time between surgery and imaging
Hollenbeck, 2003 [11]	ND	Plain X-ray (KUB)	1 month
Darabi, 2005 [12]	ND	ND	ND
El-Hefnawy, 2011 [13]	ND	Plain X-ray (KUB) and NCCT	After procedure and before discharge and 3 months
Mushtaque, 2012 [14]	Unclear	Plain X-ray (KUB)	1, 5 and 28 days
Gunlusoy, 2012 [15]	No stones	Plain X-ray (KUB), USS and IVU (in case of pelvicalyceal dilation)	1 day and 6 weeks
Huang, 2012 [16]	<1 mm	CT	7 days
Isen, 2012 [17]	<4 mm	Plain X-ray (KUB) and USS or NCCT	7 days
Atis, 2013 [18]	<4 mm	USS and IVU	1 day and 1 month
Alkan, 2014 [19]	<4 mm	NCCT or USS	3 months (stent removal at 3–4 weeks, imaging 2 months after)
Drake, 2015 [20]	<2 mm	Plain X-ray (KUB) or USS	8–12 weeks
Bansal, 2016 [21]	<4 mm	Plain X-ray (KUB), USS or CT	ND

*ND* not documented, *NCCT* non-contrast CT scan, *USS* ultrasound scan

### Post-Operative Characteristics and Patient Outcomes

1. Operative time:
  Overall mean operative time was 68.7 min (SD = ±15.2 min), although two studies did not report operative times [12, 13].
2. Proportion of patient requiring insertion of ureteric stent
  Reporting on post-operative stent insertion was variable. Ten studies reported on post-operative ureteric stent insertion, and of those only two studies reported whether they were inserted bilaterally or unilaterally [18, 20]. The study by Darabi et al. [12] was unclear on their results on stent insertion (see Table 4). The overall stent insertion was 88.8%. Of the two studies that reported on bilateral stent insertion the overall bilateral stent insertion rate was 71.6%.
3. Stone-free status (SFR)
  All studies reported on SFR, with a mean initial and final SFR of 87 and 92% respectively [11–21]. The overall ureteric SFR was 94.0% and the overall renal SFR was 81.0%.
4. Hospital stay
  Eight studies [13–15, 17–21] reported on hospital stay, with an overall mean hospital stay of 1.6 days (SD = ±0.53) (see Table 6).

**Table 6:** Patient outcomes

Study	Mean hospital stay, days (range)	Initial (%)	Final (%)
Hollenbeck, 2003 [11]	ND	ND	88.0
Darabi, 2005 [12]	ND	84.2	84.2
El-Hefnawy, 2011 [13]	2.3 ± 1 (1.5–7)	86.0	95.5
Mushtaque, 2012 [14]	2.35 (1–5)	85.0	85.0
Gunlusoy, 2012 [15]	2.4 ± 0.9 (1–5)	90.0	94.5
Huang, 2012 [16]	ND	ND	ND
Isen, 2012 [17]	1.2 (1–3)	90.2	98.7
Atis, 2013 [18]	1.37 (±0.72)	92.8	97.6
Alkan, 2014 [19]	1.23 (±0.57)	86.3	88.6
Drake, 2015 [20]	0.9 (0–7)	80.0	92.8
Bansal, 2016 [21]	1.37 (±0.72)	87.0	97.3

*ND* not documented, *SFR* stone-free rate
5. Complication rates (Table 7)

**Table 7:** Complications during periods 1 and 2, period 1 vs period 2, Clavien I–II (*p* < 0.001), Clavien ≥ III (*p* < 0.001)

Study period	Clavien I–II (%)	Clavien ≥ III (%)
Period 1	Haematuria not requiring blood transfusion (10.6)	Ureteral perforation/laceration (4.2)
2003–2012	LUTS (8.7)	Mucosal injury (3.5)
	Pain requiring analgesia (8.3)	Stone migration (1.6)
	Post-operative fever (4.2)	Stent symptoms requiring early removal (0.6)
	UTI/urosepsis/pyelonephritis (2.9)	Urinoma (0.3)
	Post-obstructive diuresis (1.0)	Pulmonary embolus leading to death (0.3)
	Perinephric haematoma (0.3)	
Total	36%	10.50%
Period 2	Haematuria not requiring blood transfusion (3.4)	Stent symptoms requiring early removal (1.1)
2013–2016	Pain requiring analgesia (2.8)	
	Post-operative fever (2.8)	
	Not specified (1.1)	
	Pyelonephritis (0.6)	
Total	10.70%	1.10%

*LUTS* lower urinary tract symptoms
  All studies reported on complications, with an overall complication rate of 25.6% (*n* = 118). There were a total of 132 complications graded Clavien I–II and 35 complications graded Clavien ≥ III. One paper did not specify what the post-operative complications were [18]. One patient died as a result of a pulmonary embolus after a prolonged operation (175 min) in the study by Hollenbeck et al. [11].

### Secondary Outcomes

**Fig. 2:**
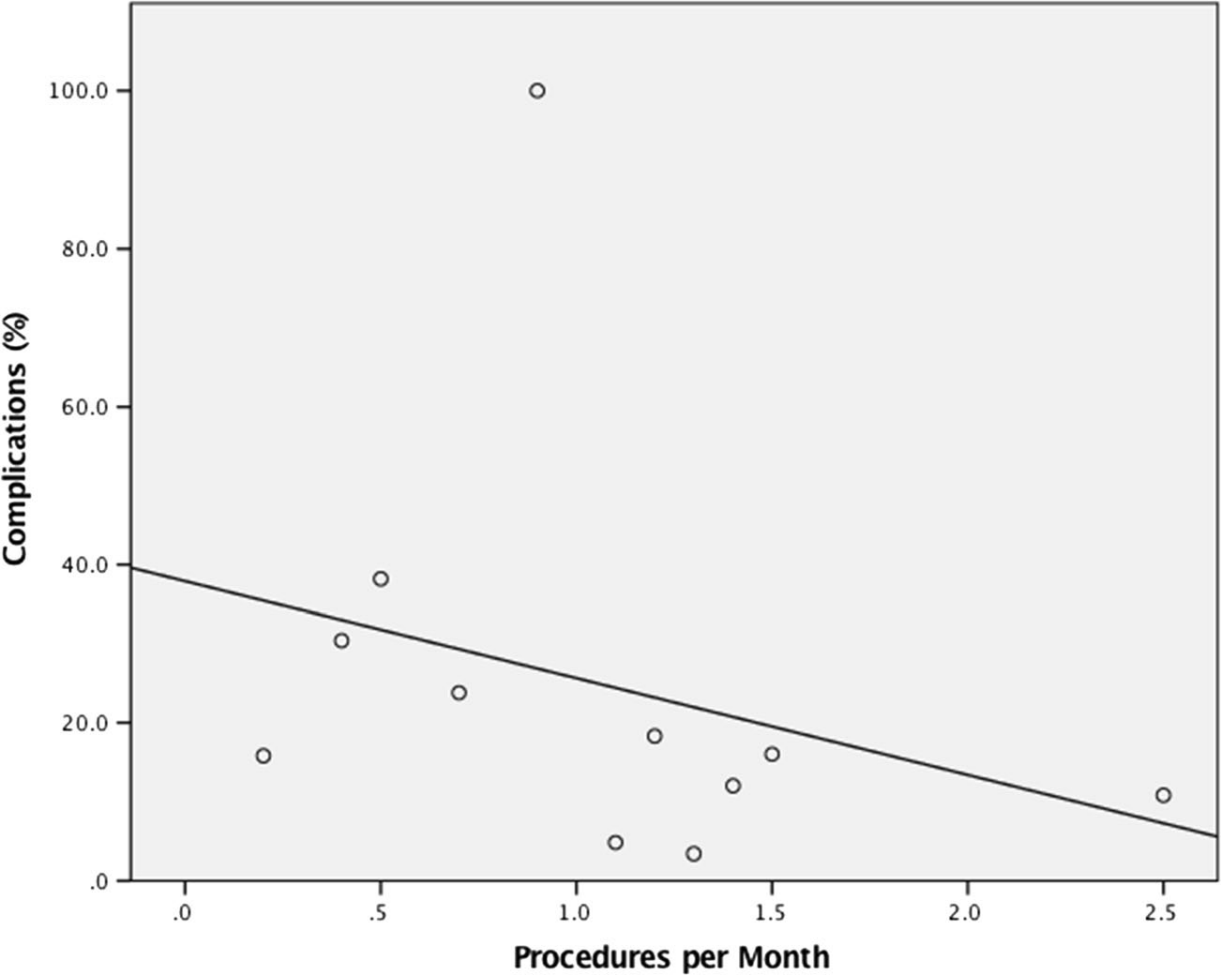
Graph demonstrating case volume (procedures per month) against post-operative complication rate. Significant regression, *p* = 0.045, *B* = −0.285, *t* = 0.894, 95% CI = 1.156 to 75.602
*Stone size*: There were no other significant correlations between complication rate and stone size (*p* = 0.16).
*Holmium laser* vs. *pneumatic lithotripsy:* There were six studies [11, 16, 18–21] examining holmium laser (HL) lithotripsy and three [14, 15, 17] examining pneumatic lithotripsy (PL). There were significantly more complications after PL than HL (54.9 vs. 16.7%, *p* = 0.007, 95% CI = 0.74 to 75.83).

1. Comparison of historical versus contemporary studies
  There was no overall difference in SFR for initial and final SFR between these two time periods. Analysis of the periods 1 and 2 demonstrated a significantly longer hospital stay in period 1 (1.9 days) than in period 2 (1.3 days) (*p* = 0.034, 95% CI = 0.09 to 1.60). There were significantly more complications in period 1 (40.5%) than period 2 (11.8%) (*p* < 0.001). Sub-analysis demonstrated significantly more Clavien I–II complications (*p* < 0.001) and Clavien ≥ III (*p* < 0.001) in period 1 than period 2 (see Table 7). There was just one Clavien III complication in period 2, which was early stent removal due to stent symptoms.
2. Predictors of complications:
*Case volume*: Upon regression analysis there was a significant negative association between complication rate and case volume (procedures per month) (*p* = 0.045, *B* = −0.285, *t* = 0.894, 95% CI = 1.156 to 75.602) (see Fig. 2).

## Discussion

### Findings of Our Study

**Fig. 3:**
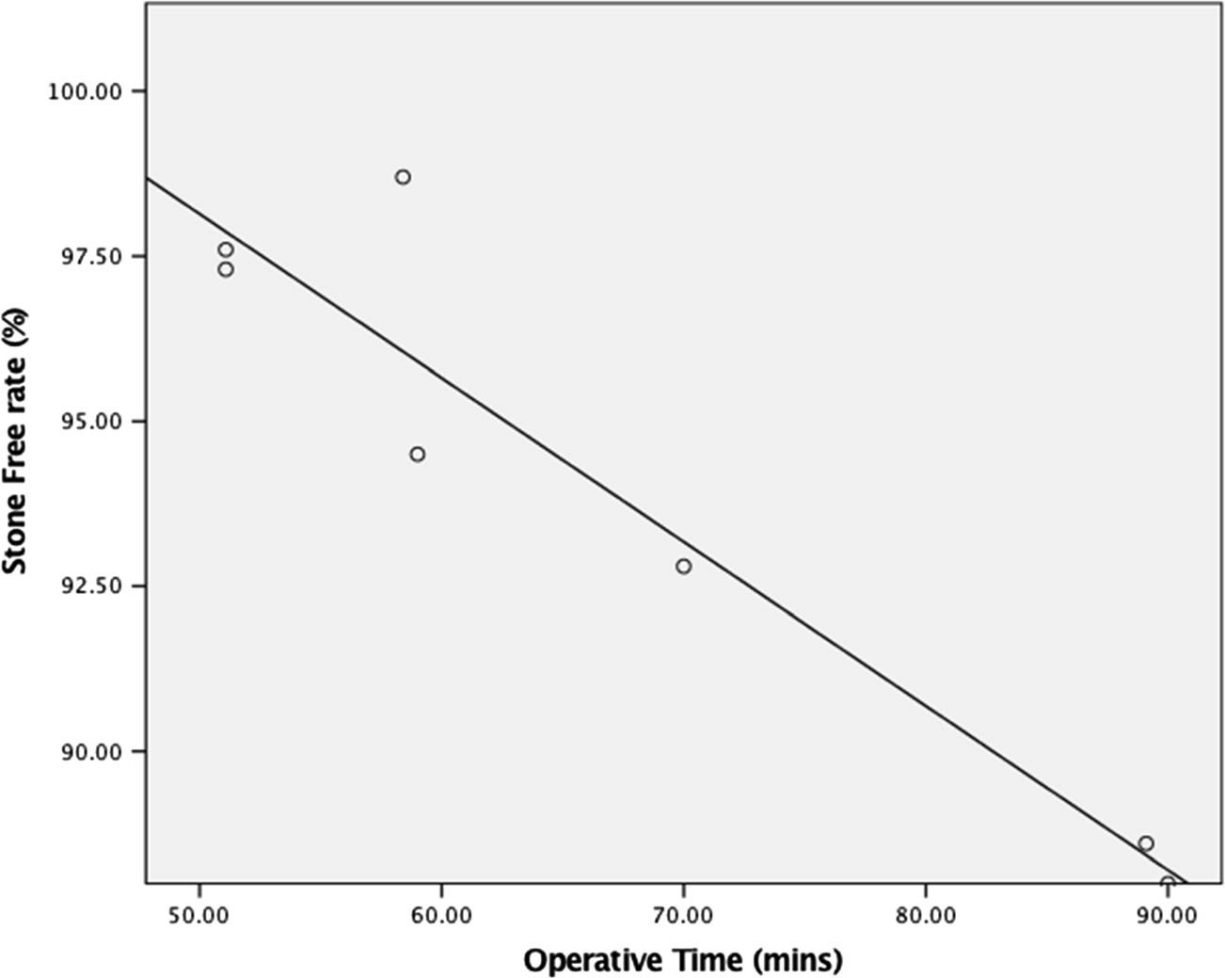
Graph demonstrating operative time (minutes) against stone-free rate. Significant regression, *p* = 0.002, *B* = −0.954, *t* = −7.81, 95% CI = −0.343 to −0.179

In this updated review, a BS-URS approach achieved an overall SFR and complication rate of 92 and 26%, respectively. The mean hospital stay was just under 2 days. Notably, hospital stay and complications are significantly reduced in the contemporary data (from 2013) when compared with historical cohorts (prior to 2013) [22••] (see Fig. 3). Furthermore, the complication rates reported in this review are nearly half those reported in the systematic review by Rai et al. [22••] This data suggest an improving trend of outcomes following BS-URS. Other factors that have significantly improved outcomes are higher case volume per surgeon and the use of holmium laser fragmentation. This is a clear reflection of evolving expertise, endoscopic laser technology and a wider variety of available technique and technology [23–30].

### Meaning and Weakness of the Study

Hollenbeck et al. [11] compared staged URS with BS-URS for bilateral urolithiasis in a retrospective case series. They reported that BS-URS was aassociated with added morbidity; however, the cumulative risk with staged URS procedures (14% per procedure) was similar to BS-URS (29%). However, this data has to be viewed with caution owing to its historical nature. Contemporary data from this review suggests a more encouraging trend with regards to complications from BS-URS. There has been a general reluctance to take up bilateral simultaneous management of urolithiasis. In a prospective study, Shen et al. [31] compared single-staged simultaneous URS and PCNL with staged procedures. They demonstrated a SFR of 92.3% with a low complication rate (11.5%). No complications were graded higher than Clavien III. Additionally, they reported significant reductions in anaesthetic time, operative time, hospital stay and costs with a single-stage approach, thus demonstrating feasibility and potential benefits of single-session strategy. Furthermore, bilateral single-session strategy has been reported with percutaneous nephrolithotomy (PCNL) and shock wave lithotripsy (SWL). The reported SFR for PCNL and SWL when employing a bilateral single-session treatment ranged between 87–96% and 60–80% [26–30], respectively. Whilst the SFR rates between PCNL and URS are similar, they have a demonstrably higher SFR in comparison to SWL. However, the complication rates with BS-PCNL range between 17 and 36% [26, 27, 30], much higher than complication rates reported in more contemporary BS-URS cohorts (period 2, complication rate of 12%). BS-URS appears a more effective and safer strategy in comparison with its other counterparts.

Superior outcomes in high-volume centers and increasing caseload have been demonstrated procedures such as URS [24] and PCNL [25]. This systematic review is the first study to corroborate this observation in the context of BS-URS. This review also demonstrated that complication rates were significantly lower in studies that employed holmium laser fragmentation. These results corroborates with finding of a previous study for impacted ureteric stones, which also revealed a higher SFR with laser lithotripsy [32].

### Areas of Future Research

Our review highlights the lack of high quality evidence on BS-URS in the management of urolithiasis, with all studies of evidence level three. With the arrival of new digital ureteroscopes, these outcomes are likely to improve further [33]. The review raises the issue of standardization of study parameters in order to make appropriate comparisons. For example, the SFR definition varied from study to study, as did the modality of post-operative imaging and time between the intervention and imaging. Due to this lack of standardization, the authors recommend interpreting the data in this study with caution. Further prospective, multi-centred studies with standardized SFR, imaging modality and outcome parameters should be conducted in this field [34].

## Conclusions

The complication rate and hospital stay for bilateral simultaneous ureteroscopy for urolithiasis are significantly reduced in contemporary studies compared to previous studies. Higher case volume and holmium laser lithotripsy are associated with fewer complications.
